# Short-term outcomes in mesh versus suture-only treatment of burst abdomen: a case-series from a university hospital

**DOI:** 10.1007/s10029-025-03279-x

**Published:** 2025-02-18

**Authors:** Thomas Korgaard Jensen, Madeline Kvist, Merete Berthu Damkjær, Jakob Burcharth

**Affiliations:** 1 Department of Gastrointestinal and Hepatic Diseases, Copenhagen University Hospital – Herlev and Gentofte, Copenhagen, Denmark; 2 Emergency Surgery Research Group Copenhagen (EMERGE Cph.), Copenhagen University Hospital - Herlev and Gentofte, Copenhagen, Denmark; 3 Department of Clinical Medicine, University of Copenhagen, Copenhagen, Denmark

**Keywords:** Laparotomy, Surgical wound dehiscence, Acute care surgery, Surgical outcomes, Surgical mesh, Incisional hernia

## Abstract

**Purpose:**

Surgery for a burst abdomen after midline laparotomy is associated with later incisional hernia formation. Accommodating prophylactic measures, notably mesh augmentation, are of interest. However, data regarding safety and outcomes are scarce. This study aimed to evaluate the short-term risk profile of mesh prophylaxis in the context of a burst abdomen.

**Methods:**

This is a single-center prospective study of patients suffering from burst abdomen from 2021 to 2023. A treatment protocol for the management of burst abdomen was introduced, including the synthetic, partially absorbable onlay mesh. Adult patients (≥ 18 years) with a life expectancy of > 1 year with no plans of future pregnancies were recommended to be treated with a prophylactic mesh. In this analysis, adult patients were included if they suffered from a burst abdomen after elective or emergency laparotomy. The study evaluates short-term outcomes, including 90-day wound complications, length of stay, and mortality.

**Results:**

Sixty-seven patients fulfilled the inclusion criteria and underwent treatment for a burst abdomen during the study period. Thirty-eight patients were treated with a suture-only technique, and 29 patients were supplemented with a mesh. 13 of 14 observed wound complications in the mesh group were of mild degree (Clavien Dindo 1-3b), while one patient (3%) needed mesh-explantation. The 90-day mortality rate was 21% and comparable between suture-only and mesh techniques.

**Conclusion:**

Mesh augmentation in surgery for a burst abdomen seems safe in well-selected patients at 90 days follow-up. Long-term data on the prophylactic effect on hernia development is needed.

**Supplementary Information:**

The online version contains supplementary material available at 10.1007/s10029-025-03279-x.

## Background

In midline laparotomy, a burst abdomen is a feared complication due to significant increases in patient morbidity and mortality [1–4]. A rising number of studies and guidelines have sought to identify prophylactic interventions to reduce the incidence of burst abdomen, however, with diverging results [5–11]. As such, rates of burst abdomen after midline laparotomy are reported to be 0–14% [11, 12].

The best approach to managing a burst abdomen remains inadequately defined due to sparse evidence [3]. Associated outcomes, including short-term issues such as wound complications and recurrent burst abdomen and long-term challenges such as incisional hernia development and diminished quality of life, are well known [1, 2, 13–15]. Incisional hernia formation has been reported in up to 83% of patients after a burst abdomen [14, 16–21]. Patients with incisional hernias often require complicated surgical procedures with the affiliated risk of chronic pain, reduced physical performance, low quality of life, and a notable risk of recurrence, necessitating further surgical hernia repair [22–25].

Mesh augmentation is arguably the one measure that could accommodate the risk of incisional hernia formation. Yet, the use of prophylactic meshes, especially in emergency surgery, carries the worry of mesh-related complications and has kept the introduction at bay [3, 26]. Within the last decade, a rising number of studies have suggested that prophylactic mesh augmentation in emergency laparotomy might be safe and probably deliver a long-term increase in hernia-free survival. Although surgical site occurrences seems to increase with mesh application in the emergency laparotomy, reported in up to 20.6% of the cases, partial or total mesh removal is only reported in few studies with a rate of 2.8-3.2% [12, 14, 16–20, 27]. However, data are scarce, and methods and outcomes have been heterogeneous [28].

We hypothesized that mesh augmentation in surgery for a burst abdomen is safe in selected patients. This study aims to report the 90-day outcomes of patients treated for burst abdomen with or without a prophylactic mesh as a supplement.

## Methods

This single-center cohort study was based on prospective registration of patients undergoing emergency surgery in a local database. The study was approved by the Danish Data Protection Agency and the capital region of Denmark (P-2020-1166, R-21038079, P-2921-533). As it was purely observational, without any intervention or randomization of patients, the Danish Research Ethics Committee did not require approval. The study was reported by the Reporting of Observational Studies in Epidemiology (STROBE) statement [29].

### Setting

The study was conducted in the surgical section of the Department of Gastrointestinal- and Hepatic Diseases, Copenhagen University Hospital Herlev, a university hospital treating emergency admittances from an area covering 465,000 patients. The department consists of subspecialized surgical teams, including an emergency surgical team solely performing emergency surgical and endoscopic procedures. The department has a well-described strategy towards high-risk emergency surgical patients, including standardized pre- [30], intra- [31], and postoperative [32] frameworks, including documented standards for abdominal wall closure [11], the use of open abdomen strategies [33, 34], and surgical treatment of burst abdomen [2].

## Data collection and management

Data was recorded prospectively until discharge in all patients undergoing emergency laparotomy due to major pathology, including bowel obstructions, bowel perforations, ischemia, and intraperitoneal bleedings, as well as re-operations of both emergency- and elective cases. Data was collected via an electronic journal system (EPIC Hyperspace, Epic Systems Corp 2020) and stored in a REDCap database [35].

The database includes preoperative data on demographics, comorbidities, height, weight, tobacco- and alcohol consumption, American Association of Anesthesiologists (ASA) score, and World Health Organization (WHO) performance status. Intraoperative data included indication for surgery, type of procedure (both for index procedures and later re-operations), and specific documentation for abdominal wall closure. Peritoneal contamination was classified by the Center for Disease Control and Prevention (CDC) classification scale (1: clean, 2: clean-contaminated, 3: contaminated, and 4: dirty) [36]. Postoperative collected data consists of medical and surgical complications, including specific data collection on wound complications and postoperative death. Complications are scored by the Clavien-Dindo (CD) system for grading complications [37].

### Participants and outcomes

All adult patients (≥ 18 years) undergoing surgery for a burst abdomen after midline laparotomy from the 1st of January 2021 to the 31st of December 2023 were evaluated.

Burst abdomen was defined as acute rupture of the sutured midline aponeurosis and was diagnosed either by visual inspection of the wound with visible bowel and/or omentum in the wound or, without concomitant skin-rupture, by re-opening of the skin-closure on the suspicion, typically caused by large amounts of fluid discharge through the skin closure [2, 3].

The primary outcome was the frequency of overall wound complications in the first 90 days after surgery for a burst abdomen, stratified by mesh augmentation in midline laparotomies. Secondary outcomes were rates of specific wound complications, including the need for mesh explantation, length of stay, and mortality rates.

### Identification, registration, and classification of wound complications

Identification and registration of wound complications were performed by senior emergency surgeons, who managed the daily ward rounds for these patients. Data was recorded prospectively until discharge, and retrospectively from discharge until the 90th postoperative day. We defined short-term wound complications as any surgical site occurrence [38] within the first 90 days after surgery for a burst abdomen, including:

- *Superficial infection*, being an infection involving only the skin and subcutaneous tissue, and diagnosed by either discharge of pus from the wound without visualization of the sutured aponeurosis and/or mesh or treatment for a clinically diagnosed superficial wound infection [39].
- *Deep infection*, being an infection involving the deep soft tissues of the incision including the sutured aponeurosis and/or mesh, and diagnosed by pus, an abscess or other evidence of infection [39].
- *Hematoma* and *Seroma*, diagnosed either upon re-opening of the wound, by clinical diagnosis, or by radiologic imaging.
- *Superficial wound dehiscence*, diagnosed upon wound inspection as defects of the skin-closure after removal of sutures/staples without any signs of the above-mentioned wound complications.
- *Re-dehiscence*, being a recurrence of burst abdomen after surgical treatment for this complication and is otherwise defined as above.

### Abdominal wall closure and the strategy for treatment of burst abdomen

The department has had continuously updated written standards since 2016 for both abdominal wall closure after primary laparotomy [11] and handling of burst abdomen [2], (Appendix A). Our center coheres to stringent indications for the use of open abdomen therapy as recommended by the World Society of Emergency Surgery [40] and has been previously described [34](Appendix B).

*Primary abdominal wall closure* is performed with a 150 cm long, slowly absorbable polydioxanone suture size 2 − 0 mounted on a 28 mm needle, taking small 5–9 mm bites only of the midline aponeurosis, and suturing continuously with 5 mm between stitches resulting in a suture length to wound length ratio (SL: WL) of at least 4:1 [11].

**Fig. 1:**
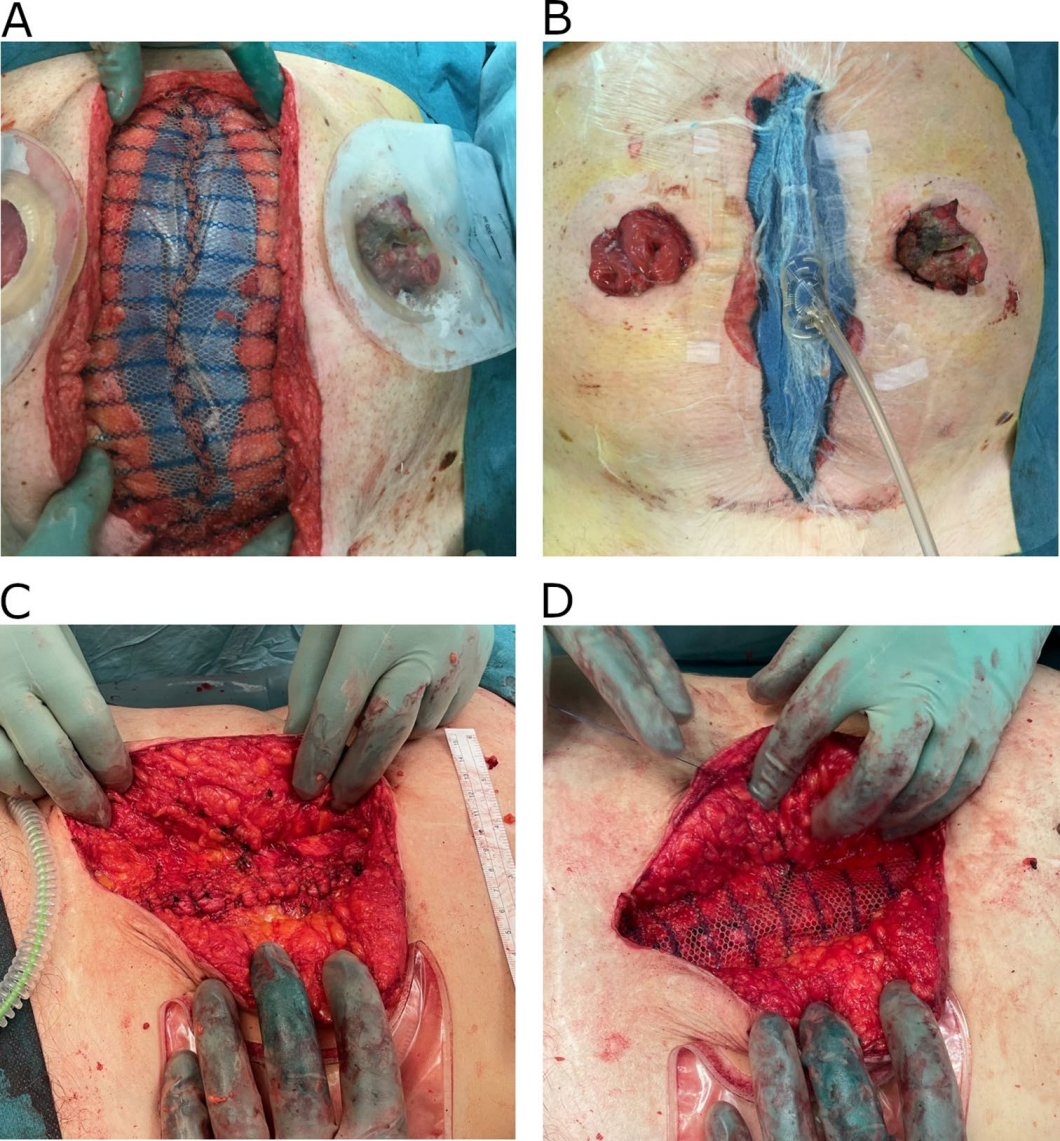
Two patients treated for burst abdomen. With large intraabdominal volume and/or loss of fascial domain, intraabdominal draping, mesh-mediated traction (**A**) and negative pressure therapy (**B**) was applied to the wound. When fascial closure was possible, mass-closure continuous suturing was used (**C**). If no contraindications were identified, an on-lay synthetic, partial absorbable mesh was augmented (**D**)

*Abdominal wall closure in the context of a burst abdomen* is performed with a 150 cm long, slowly absorbable polydioxanone suture size 0 on a large CTX needle taking 3 cm large mass-closure bites of all layers (subcutaneous fat and skin excluded) continuously, with small steps of 5 mm between each stitch, resulting in a SL: WL of at least 10:1 (Fig. 1) [2].

*The choice to initiate an open abdomen strategy* as opposed to abdominal wall closure is only chosen in the clinical situations of (a) persistent and severe unstable physiology (hypotension, hypothermia, pH < 7.20 or coagulopathy), (b) the need for reassessment of bowel viability, (c) loss of abdominal wall domain and/or too much tension needed for aponeurotic adaptation, and (d) in the context of abdominal compartment despite all non-operative actions been initiated (full medical muscle-relaxation, drainage of fluid-collection, decompression of the stomach with a tube and the urinary bladder with catheter) [34].

*Techniques for open abdomen, mesh-mediated fascial traction, and timing of fascial closure* included using a generic system for temporary abdominal closure, utilizing the Abthera (3 M) protective plastic dressing, and application of 125mmHg continuous negative pressure. Mesh-mediated fascial traction was applied if fascial closure was impossible at the first change of the abdominal dressing (Fig. 1). The mesh was usually removed when fascial closure was possible, and a new permanent mesh would be applied if indicated. Changes of dressings were done three times a week until a tension-free fascial closure was possible. If severe fibrotic bowel adherences to fascia or subcutaneous fat were identified, predicting a high risk of bowel injury and entero-atmospheric fistula formation, fascial closure was abandoned resulting in skin-only closure with the acceptance of a formation of an incisional hernia [33, 34].

*Application of prophylactic mesh* in patients treated for burst abdomen was recommended under certain circumstances, being adult patients (≥ 18 years) with a life expectancy of > 1 year, with no plans of future pregnancies. Wound contamination was only a relative contraindication, but in the context of suspected contamination, a subcutaneous vacuum dressing was applied, with changes of this dressing two times a week bedside until a clean wound and initiation of granulation through the mesh was allowing for skin-closure in local anesthesia. The mesh was placed onlay with a 3 cm overlap both laterally, cranially, and caudally, and was sutured to the fascia at its border, with single stitches of a non-absorbable, monofilamentous suture, size 2 − 0 with approximately 2 cm between stitches (Fig. 1). Mesh-type for both mesh-mediated traction and prophylactic onlay placement was a macroporous, partially absorbable synthetic mesh (Ultrapro, Ethicon).

### Statistical analysis

Continuous data is presented as means with standard deviation (SD). Categorical data is presented as frequencies and percentages. Data distribution was assessed by visual inspection of histograms and QQ plots. Univariate analysis of categorical data was performed utilizing the Pearson χ2 test unless less than five observations were done, in which case Fisher’s exact test was applied. The mean of numerical data was compared using an independent t-test. All p-values are presented as two-sided, and values of ≤ 0.05 were considered to be statistically significant.

## Results

**Fig. 2:**
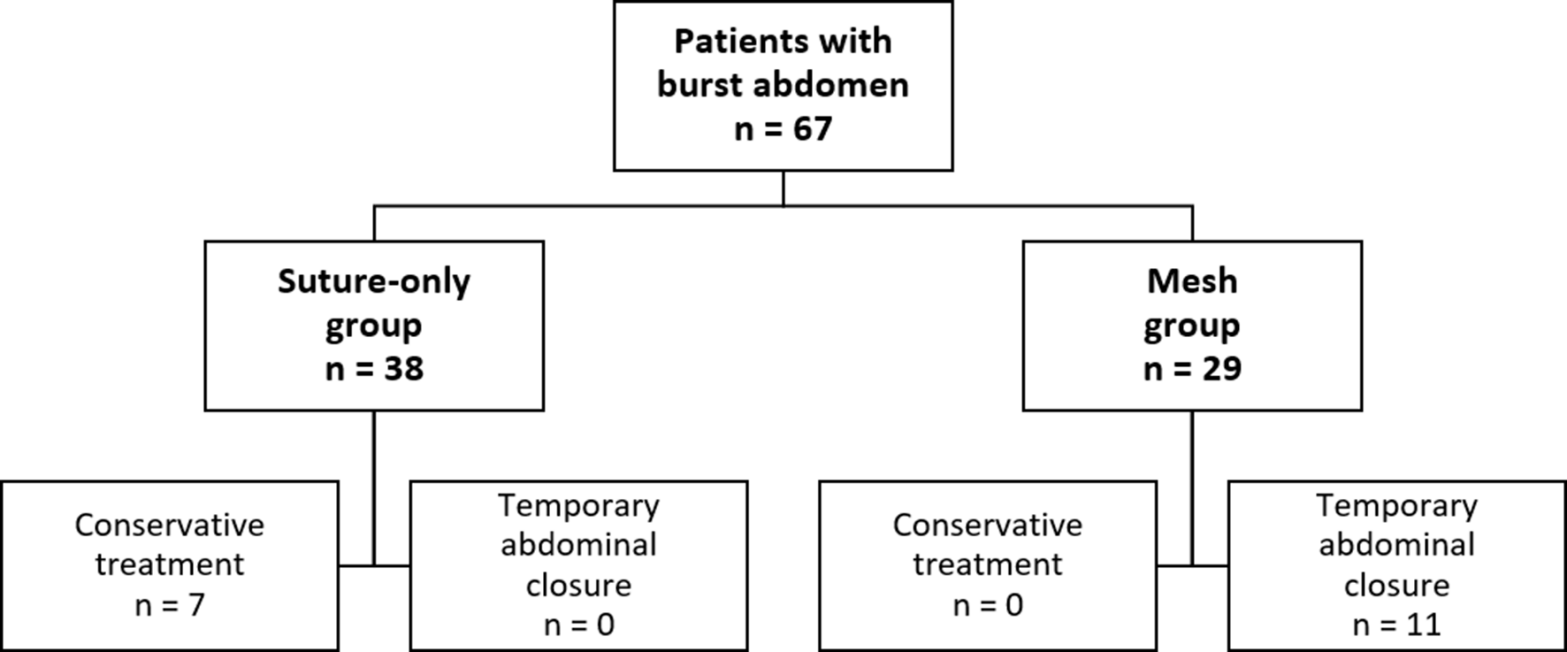
Flowchartof the study population and groups including patients treated consevartively for a burst abdomen (only skin-closure) and patientents with a course of temporaty abdominal closure before fascial closure

Preoperative data is outlined in Table 1. Through the period, a total of 67 patients were identified with a burst abdomen after midline laparotomy. 47 patients (70%) were male. 38 patients (56%) were treated without a permanent mesh (suture-only group), and 29 patients (44%) were treated with a permanent mesh (mesh group) (Fig. 2). Only a few female patients were treated with a mesh, with 25 patients (86%) of the patients in the mesh group being males (*p* = 0.012). No differences were identified between the two groups regarding ASA score, WHO performance status, tobacco use, or alcohol consumption. More patients in the mesh group (9 of 29 patients, 31%) had a Body Mass Index of 30 kg/m^2^ or higher compared to patients in the suture-only group (5 of 38 patients, 13%; *p*=0.075). There was no difference in the characteristics of the index procedure before the event of a burst abdomen between the two groups, with 90% in the suture-only group vs. 79% in the mesh group being an emergency procedure (*p*=0.274). 28 of 67 patients (42%) had peritonitis at index surgery with no differences between the two groups.

**Table 1:** Baseline characteristics of patients treated for a burst abdomen with a suture-only technique or with supplemental mesh

Burst Abdomen *N*, total = 67(%)						
		Suture only		Mesh		
		*n* = 38		*n* = 29		*P*
Sex, Male		22	(58)	25	(86)	0.012
Age, years	< 60	4	(11)	7	(24)	.322^a^
	60–80	27	(71)	18	(62)	
	> 80	7	(18)	4	(14)	
ASA-score	≥ 3	14	(47)	14	(48)	0.347
WHO Performance status	≥ 3	5	(13)	5	(13)	0.974
Body Mass Index	≥ 30 kg/ m^2^	5	(13)	9	(31)	0.075
Active smoking^b^		9	(24)	8	(28)	0.764
Weekly alcohol consumption	> 10 units	12	(32)	10	(35)	0.861
Previous midline laparotomy		8	(21)	4	(14)	0.334
**Co-morbidities**	Malignancy	14	(37)	14	(48)	0.347
	Cerebro-vascular^c^	2	(5)	0	(0)	.318^a^
	Diabetes	4	(11)	1	(3)	.274^a^
	Cardiac^d^	14	(37)	14	(48)	0.347
	COPD	3	(8)	3	(10)	.526^a^
	Chronic Renal Failure	1	(3)	1	(3)	.682^a^
	Liver cirrhosis	2	(5)	0	(0)	.318^a^
**Characteristics of index procedure**	Emergency procedure	34	(90)	23	(79)	0.274
	Gastro-duodenal	2	(5)	0	(0)	.152^a^
	Adhesiolysis	7	(18)	1	(3)	
	Small bowel surgery^e^	12	(32)	10	(35)	
	Large bowel surgery^e^	17	(45)	18	(62)	
Contamination	Peritonitis^f^	18	(47)	10	(35)	0.289
	CDC grade I	3	(8)	6	(21)	0.303
	CDC grade II	17	(45)	13	(45)	
	CDC grade III	7	(18)	2	(7)	
	CDC grade IV	11	(29)	8	(27)	

ASA, American Association of Anesthesiologist; WHO, World Health Organization; COPD, Chronic Obstructive Pulmonary Disease; CDC, Center for Disease Control and prevention

^a^ Fisher’s exact test

^b^ use of tobacco-products within 8 weeks up to admittance

^c^ ischemic or hemorrhagic stroke

^d^ atrial fibrillation, chronic ischemic heart-disease, chronic cardiac failure, or essential arterial hypertension

^e^ bowel resection, enterotomies and/or stoma creation

^f^ peritonitis defined as CDC wound classification Grade III + IV

### Surgical treatment of burst abdomen

**Fig. 3:**
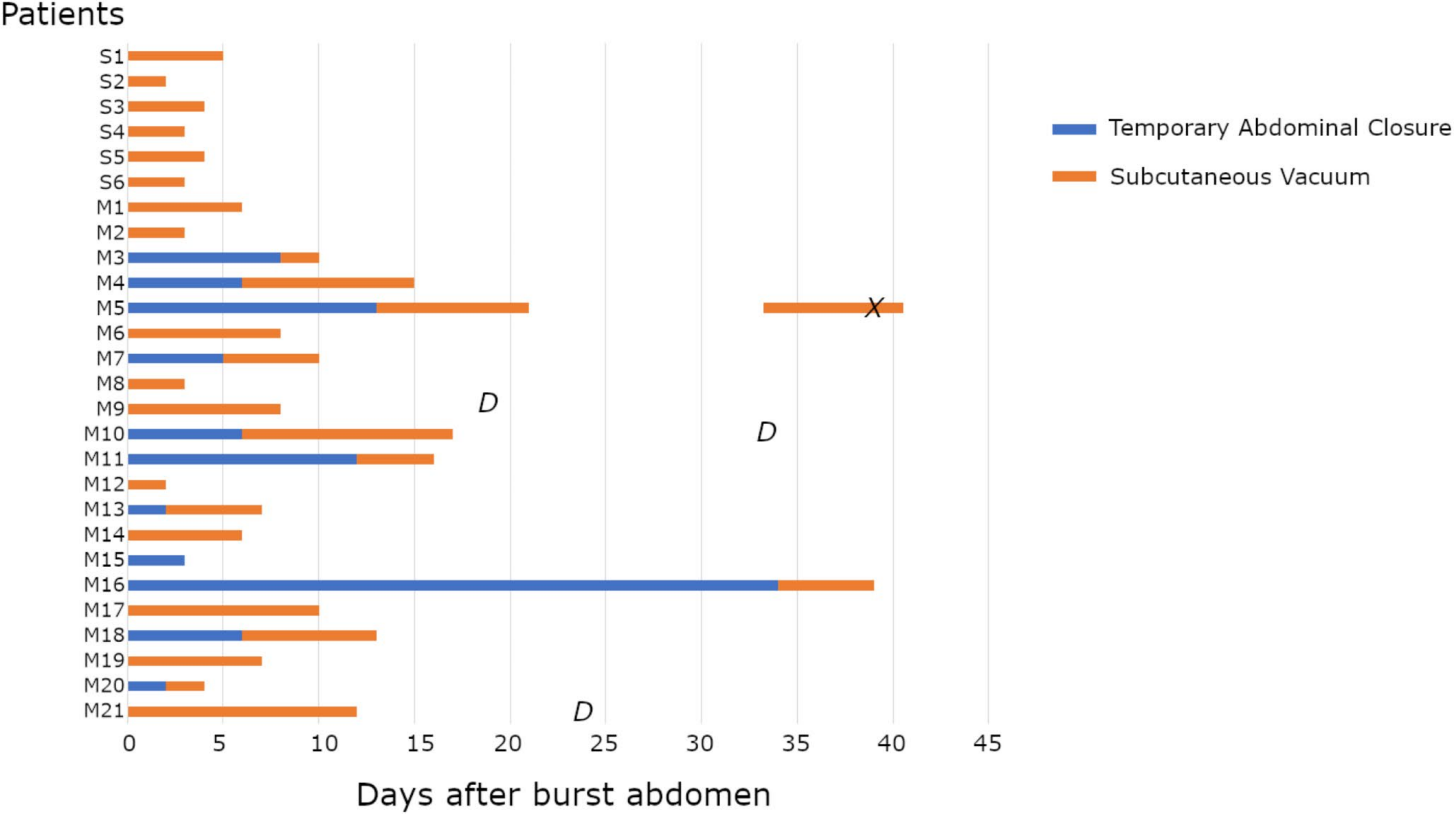
Specific course and outcomes of the patients treated for burst abdomen with the need of temporary abdominal closure and/or subcutaneous vacuum treatment. Patients not included In the figure: 32 patients in suture-only group and 8 patients in mesh-group with immediate fascial and skin-closure.Sx, Patients within the suture-only group; Mx, Patients within the mesh-group; X, Mesh explantation; D, Death

Perioperative data from the surgery for burst abdomen is outlined in Table 2, and patients not having their fascia and/or skin closed at first re-operation are further schematized in Fig. 3. In the event of a burst abdomen, 13 of the 38 patients (34%) in the suture-only group suffered from peritonitis compared to four of the 29 patients (14%) in the mesh group (*p* = 0.051). Two of these four patients suffered from severe peritonitis (CDC grade IV). In the suture-only group, seven of 38 patients (18%) were treated without fascial closure, and only skin closure were performed. In six patients, this was due to severe fibrotic adherences, and in one patient, an entero-atmospheric fistula formed. In the last patient, fascial closure was impossible due to severe loss of domain in a patient deemed too frail to go through a series of operations with a prolonged open abdomen. All patients in the suture-only group, except patients who underwent conservative treatment, had their fascia closed at the first operation for a burst abdomen, while a mean delay of 3.3 days in the mesh group was seen (*p* = 0.01). Hence, temporary abdominal closure was only applied among patients in the mesh group (11 of 29 patients, 38%, *p* < 0.001). The mean SL: WL was comparable between the two groups (13:1 in the suture-only group vs. 14:1 in the mesh group, *p* = 0.39). All patients with delayed skin closure were treated with subcutaneous vacuum therapy; six of the 38 patients (16%) in the suture-only group vs. 20 of the 29 patients (69%) in the mesh group (*p* < 0.001).

**Table 2:** Perioperative characteristics for suture-only vs. mesh groups

Surgery for burst abdomen, intraoperative data *N*, total = 67(%)						
		Suture only		Mesh		
		*n* = 38		*n* = 29		*P*
Days from index surgery to burst abdomen	Mean (SD)	8.7	(3.3)	7.7	(3.8)	.240
Degree of contamination	Overall peritonitis^a^	13	(34)	4	(14)	.051^b^
	CDC Grade II	25	(66)	25	(87)	.162^b^
	CDC Grade III	7	(18)	2	(7)	
	CDC Grade IV	6	(16)	2	(7)	
Days to fascial closure	Mean (SD)	0	(0)	3.3	(6.9)	0.01
Days from fascial closure to skin closure	Mean (SD)	0.62	(1.4)	4.4	(3.8)	< 0.001
Conservative treatment		7	(18)	0	(0)	.015^b^
Temporary abdominal closure applied		0	(0)	11	(38)	< 0.001^b^
Subcutaneous VAC applied before skin closure		6	(16)	20	(69)	< 0.001
Suture length to wound length ratio (if fascial closure)	Mean (SD)	13	(4.5)	14	(4.1)	0.39

SD, Standard Deviation; CDC, Center for Disease Control and Prevention

^a^ Peritonitis defined as CDC wound classification grade III + IV

^b^ Fisher’s exact test

There was no difference regarding age, BMI, comorbidities, or characteristics of the primary procedure between males and females. Seven of the 20 (35%) female patients and 10 of the 47 (21%) male patients suffered from peritonitis at the time of surgery for a burst abdomen (*p* = 0.237), while only two of the 20 (10%) female patients compared to 16 out of 47 (34%) male patients underwent a course of open abdomen treatment before having the fascia closed (*p* = 0.042).

### Postoperative outcomes

Short-term wound complications after surgical treatment for a burst abdomen are outlined in Table 3. There were 36 wound complications in 67 patients, with no statistical differences between the two groups. Two recurrences of burst abdomen occurred in the non-mesh group, while no patients in the mesh group suffered from this. Within the mesh group, 14 of 29 patients (48%) suffered from a wound complication, with the majority of these being mild complications graded as Clavien-Dindo grade I (12 of 14 patients). One patient needed surgical treatment under general anesthesia (CD grade IIIb). This patient suffered from a deep wound infection 11 days after skin closure. The wound was initially reopened and treated with subcutaneous vacuum therapy aiming to obtain granulation tissue through the mesh. Yet, after six days and three dressing changes, this strategy was abandoned, and the mesh was surgically removed. The patient was discharged with a partially closed wound at the skin level and was followed on an outpatient basis, and complete secondary healing was obtained within three weeks.

**Table 3:** Comparison of short-term (90 days) wound complications after surgery for a burst abdomen in suture-only vs. mesh groups

Short term (90 days) wound complications *N*, total = 67(%)						
		Suture only		Mesh		
		*n* = 38		*n* = 29		*P*
Type of complication	Overall	22	(58)	14	(48)	.174^a^
	Hematoma	0	(0)	3	(10)	
	Seroma	4	(11)	3	(10)	
	Superficial wound dehiscence	7	(19)	5	(17)	
	Burst abdomen (recurrence)	2	(5)	0	(0)	
	Entero-atmospheric fistula	1	(3)	0	(0)	
	Superficial wound infection	8	(22)	2	(7)	
	Deep (mesh) infection and explantation	NA		1	(3)	
Clavien Dindo grade of complication	1	16	(42)	12	(41)	.511^a^
	2	1	(3)	0	(0)	
	3a	0	(0)	1	(3)	
	3b	4	(11)	1	(3)	
	4	1	(3)	0	(0)	
Length of stay, days	Mean (SD)	22	(13)	27	(13)	.08^b^
Mortality	30 days	8	(21)	3	(10)	.202^a^
	90 days	9	(24)	5	(17)	0.52

SD, Standard Deviation

^a^Fisher’s exact test

^b^ Independent T-test

The mean length of stay was 22 days in the suture-only group compared to 27 days in the mesh group (*p* = 0.08). Mortality rates were comparable between the groups, with a 90-day mortality rate of 21% (14 of 67 patients) among all patients treated for burst abdomen in the study period.

## Discussion

Between January 2021 and December 2023, we treated 67 patients for burst abdomen after midline laparotomy. To accommodate a well-known high risk of incisional hernia formation after treatment of this complication, our center initiated an option for mesh application before the study period [1, 3]. Of the 67 patients treated for burst abdomen, 29 (43%) were supplemented with a mesh. No differences in the rate or severity of wound complications between sutured-only patients and mesh-augmented patients were identified. Wound complications were common, with 53% of the patients (36 of 67 patients) suffering from any wound-related complication. However, 78% (28 of 36 complications) were minor complications with no need for surgical intervention. We registered four major surgical complications. Within the suture-only group, two patients suffered from recurrence of burst abdomen, and one patient developed an entero-atmospheric fistula. Within the mesh group, one out of 29 patients (3%) suffered from mesh infection with the need for mesh explantation which is comparable to similar studies [12, 16, 18, 19].

Risk factors for a burst abdomen are multiple and heterogeneous [2, 3, 19, 41–45]. In our study, identifying risk factors for a burst abdomen was not possible due to the study design. However, we found that patients suffering from burst abdomen were often male (47 of 67 patients, 70%), had a high ASA score ≥ 3 (28 of 67 patients, 42%), and often suffered from comorbidities, including malignant diseases. Furthermore, most patients went through an emergency procedure before the event of a burst abdomen (55 of 67 patients, 82%). These findings are comparable to the literature and probably, at least to some extent, contribute to the high 90-day mortality rate of 21% (14 out of 67 patients) [11, 46–48]. In this case series, mesh augmentation was more often chosen in case of a clean wound, obesity, and a preceding course of open abdomen. Our study is not designed to evaluate contraindications for mesh augmentation in detail, and although wound contamination was not an absolute contraindication, it seems reasonable that surgeons are cautious in case of a contaminated wound, knowing there is a risk of mesh infection [3, 26]. Obesity is a well-known risk factor for incisional hernia formation and, in combination with a burst abdomen, probably leaves a patient at high risk [49–51]. We apply temporary abdominal closure whenever fascial adaptation is not possible due to excessive retraction of the fascia or increased abdominal volume, and only patients that underwent this treatment also received a mesh (0% in the suture-only group vs. 38% in the mesh group, *p* < 0.001). Patients undergoing open abdomen treatment potentially are more physiologically deranged, and edematous abdominal wall and/or abdominal wall necrosis in this context might stimulate the surgeon to apply a mesh based on an expectation that the risk of incisional hernia formation might be high [52, 53].

The treatment of burst abdomen is still debated and high morbidity- and mortality rates, high rates of recurrence, extended length of stay, and a remarkably high rate of incisional hernia formation are some of the challenges that need focus [1, 47]. Earlier, we documented that surgery for a burst abdomen with a suture-only strategy with a mass-closure technique produced low rates of recurrence, but the incisional hernia rate remained high at 33% with a median follow-up of 17 months (min 4, max 67 months), which is comparable to the literature [1, 2, 18, 19]. This understated the broadly recognized need for further actions toward hernia prophylaxis in patients treated for burst abdomen [3]. Prophylactic mesh augmentation has been a main topic in the debate. However, the hernia societies have not been able to clearly state when and how a surgeon should choose prophylactic mesh in laparotomies, due to only low-quality evidence [3, 26]. Mesh infection, with or without the need for mesh explantation, is a relevant and feared complication, and as of today, it is up to every surgeon and/or center to decide when prophylactic mesh should be considered. The patient presented in this study, with mesh infection and need of explantation, tolerated the complication well. However, the finding underscores the importance of the debate surrounding this type of surgical modality. Only a few studies investigate the effect of prophylactic mesh in surgery for burst abdomen [16, 18, 19]. The results suggest a reduction in incisional hernia formation compared to suture-only techniques. The studies are small and mostly of retrospective design, leaving a high risk of bias. However, the results align with the evidence in general regarding the benefits of prophylactic mesh augmentation in primary laparotomy in high-risk cases [54–56]. It is important to further investigate risk profiles and profits of mesh augmentation in surgery for burst abdomen before proper guidelines with high-quality evidence can be designed. Our study only investigates short-term outcomes and long-term follow-up on this patient-cohort will reveal if our technique presents any benefits towards incisional hernia formation.

In this case-series we introduced a new algorithm to the treatment of burst abdomen, with the option of mesh-augmentation in the case of burst abdomen, which was applied in 44% of cases. Introduction of new surgical techniques, especially ones that in general are considered associated to an increased risk-profile, potentially can be met with resistance amongst surgeons. We cannot speculate in whether 44% is an appropriate rate of patients to receive a mesh and randomized trials comparing mesh-augmentation to a suture-only technique are needed.

Our study is limited to its design and sample size. The study is a non-randomized, single-center study and data is not necessarily generalizable. With 67 patients treated for burst abdomen, and a subgroup of 29 patients receiving mesh-closure, we cannot be sure that the results are not a product of a small sample size. However, burst abdomen is a rare complication and data at least suggest that mesh augmentation in the emergency setting of burst abdomen is relatively safe. This study investigates short-term outcomes regarding wound complications and cannot comment on long-term consequences, including the risk of hernia development. Further studies are needed regarding burst abdomen prophylaxis and regarding improvement of both short-term and long-term outcomes of patients suffering from burst abdomen.

In conclusion, mesh augmented closure of burst abdomen seems to be safe in well-selected patients, producing primarily mild wound complications comparable to suture-only techniques.

## Electronic supplementary material

Below is the link to the electronic supplementary material.

Supplementary Material 1

## Data Availability

Data are not publicly available due to sensitive data but can be requested in an anonymized format by the corresponding author.

## References

[1] Jensen TK, Gögenur I, Tolstrup MB (2022) High rate of incisional hernia observed after mass closure of burst abdomen. Hernia 26(5):1267–1274. 10.1007/s10029-021-02523-4 34674087 10.1007/s10029-021-02523-4

[2] Jensen TK, Gögenur I, Tolstrup M-B (2021) Standardized Surgical primary repair for Burst Abdomen reduces the risk of Fascial Redehiscence. Ann Surg 274(6):e1115–e1118. 10.1097/sla.0000000000003766 32209894 10.1097/SLA.0000000000003766

[3] López-Cano M, García-Alamino JM, Antoniou SA et al (2018) EHS clinical guidelines on the management of the abdominal wall in the context of the open or burst abdomen. Hernia 22:921–939. 10.1007/s10029-018-1818-9 30178226 10.1007/s10029-018-1818-9

[4] Kvist M, Jensen TK, Snitkjær C, Burcharth J (2024) The clinical consequences of burst abdomen after emergency midline laparotomy: a prospective, observational cohort study. Hernia Online Ahead Print. 10.1007/s10029-024-03104-x 10.1007/s10029-024-03104-x PMC11449993 39031235

[5] Millbourn D, Cengiz Y, Israellson L (2010) Effect of stitch length on wound complications. Arch Surg 145:599. 10.1001/archsurg.2010.78 20566985 10.1001/archsurg.2010.78

[6] Harlaar JJ, Van Ramshorst GH, Jeekel H, Lange JF (2010) Effect of stitch length on wound complications. Arch Surg 145:599. 10.1001/archsurg.2010.78 20566985 10.1001/archsurg.2010.78

[7] Millbourn D, Cengiz Y, Israelsson LA (2011) Risk factors for wound complications in midline abdominal incisions related to the size of stitches. Hernia 15:261–266. 10.1007/s10029-010-0775-8 21279664 10.1007/s10029-010-0775-8

[8] Harlaar JJ, Deerenberg EB, Van Ramshorst GH et al (2011) A multicenter randomized controlled trial evaluating the effect of small stitches on the incidence of incisional hernia in midline incisions. BMC Surg 11:20. 10.1186/1471-2482-11-20 21871072 10.1186/1471-2482-11-20 PMC3182877

[9] Söderbäck H, Masood A, Leo J, Sandblom G (2022) Introduction of small stitch small bite technique: a retrospective long-term follow-up. Langenbecks Arch Surg 407(6):2527–2535. 10.1007/s00423-022-02530-8 35577975 10.1007/s00423-022-02530-8 PMC9467962

[10] Albertsmeier M, Hofmann A, Baumann P et al (2022) Effects of the short-stitch technique for midline abdominal closure: short-term results from the randomised-controlled ESTOIH trial. Hernia 26(1):87–95. 10.1007/s10029-021-02410-y 34050419 10.1007/s10029-021-02410-y PMC8881264

[11] Tolstrup MB, Watt SK, Gögenur I (2017) Reduced rate of dehiscence after implementation of a standardized fascial closure technique in patients undergoing emergency laparotomy. Ann Surg 265:821–826. 10.1097/SLA.0000000000001762 28267697 10.1097/SLA.0000000000001762

[12] Lima HVG, Rasslan R, Novo FCF et al (2020) Prevention of Fascial Dehiscence with Onlay Prophylactic Mesh in Emergency Laparotomy: a Randomized Clinical Trial. J Am Coll Surg 230(1):76–87. 10.1016/j.jamcollsurg.2019.09.010 31672681 10.1016/j.jamcollsurg.2019.09.010

[13] Denys A, Monbailliu T, Allaeys M et al (2021) Management of abdominal wound dehiscence: update of the literature and meta-analysis. Hernia 25:449–462. 10.1007/s10029-020-02294-4 32897452 10.1007/s10029-020-02294-4

[14] van Ramshorst GH, Eker HH, van der Voet JA et al (2013) Long-term outcome study in patients with abdominal wound dehiscence: a comparative study on quality of life, body image, and Incisional Hernia. J Gastrointest Surg 17:1477–1484. 10.1007/s11605-013-2233-2 23715648 10.1007/s11605-013-2233-2

[15] Abbott DE, Dumanian GA, Halverson AL (2007) Management of laparotomy wound dehiscence. Am Surg 73:1224–1227. 10.1177/000313480707301205 18186376

[16] Petersson P, Montgomery A, Petersson U (2014) Wound dehiscence: outcome comparison for sutured and mesh reconstructed patients. Hernia 18:681–689. 10.1007/s10029-014-1268-y 24916421 10.1007/s10029-014-1268-y

[17] Quassemyar (2011) Dynamic Parietal Closure: initial experience of an original Parietal Closure Procedure for treatment of Abdominal Wound Dehiscence. Arch Surg 146(6):762–764. 10.1001/archsurg.2011.112 21690458 10.1001/archsurg.2011.112

[18] Jakob MO, Spari D, Zindel J et al (2018) Prophylactic, synthetic intraperitoneal mesh Versus No Mesh Implantation in patients with Fascial Dehiscence. J Gastrointest Surg 22:2158–2166. 10.1007/s11605-018-3873-z 30039450 10.1007/s11605-018-3873-z PMC6244924

[19] López-Cano M, Pereira JA, Feliu X et al (2015) Outcome of the Use of a synthetic mesh in the repair of Burst Abdomen as compared with simple suture. Int J Clin Med 06:113–118. 10.4236/ijcm.2015.63016

[20] Gislason H, Viste A (1999) Closure of burst abdomen after major gastrointestinal operations- comparison of different surgical techniques and later development of incisional hernia. Eur J Surg 165:958–961. 10.1080/110241599750008071 10574104 10.1080/110241599750008071

[21] Van ’t Riet M, Steenwijk P, Bonjer H et al (2004) Incisional hernia after repair of wound dehiscence: incidence and risk factors. Am Surg 70:281–286 15098775

[22] Van Ramshorst GH, Eker HH, Hop WCJ et al (2012) Impact of incisional hernia on health-related quality of life and body image: a prospective cohort study. Am J Surg 204:144–150. 10.1016/j.amjsurg.2012.01.012 22579232 10.1016/j.amjsurg.2012.01.012

[23] Helgstrand F, Rosenberg J, Kehlet H et al (2012) Reoperation versus clinical recurrence rate after ventral hernia repair. Ann Surg 256:955–958. 10.1097/SLA.0b013e318254f5b9 22580941 10.1097/SLA.0b013e318254f5b9

[24] Burger JWA, Luijendijk RW, Hop WCJ et al (2004) Long-term follow-up of a randomized controlled trial of suture versus mesh repair of incisional hernia. Ann Surg 240:578–585. 10.1097/01.sla.0000141193.08524.e7 15383785 10.1097/01.sla.0000141193.08524.e7 PMC1356459

[25] Flum DR, Horvath K, Koepsell T (2003) Have outcomes of Incisional Hernia Repair Improved with Time? Ann Surg 237:129–135. 10.1097/00000658-200301000-00018 12496540 10.1097/00000658-200301000-00018 PMC1513979

[26] Deerenberg EB, Henriksen NA, Antoniou GA et al (2022) Updated guideline for closure of abdominal wall incisions from the European and American Hernia societies. Br J Surg 109:1239–1250. 10.1093/bjs/znac302 36026550 10.1093/bjs/znac302 PMC10364727

[27] Burns F, Heywood E, Challand C, Lee M (2020) Is there a role for prophylactic mesh in abdominal wall closure after emergency laparotomy? A systematic review and meta-analysis. Hernia 24(3):441–447. 10.1007/s10029-019-02060-1 31641872 10.1007/s10029-019-02060-1 PMC7210219

[28] Jakob MO, Haltmeier T, Candinas D, Beldi G (2020) Biologic mesh implantation is associated with serious abdominal wall complications in patients undergoing emergency abdominal surgery: a randomized-controlled clinical trial. J Trauma Acute Care Surg 89:1149–1155. 10.1097/TA.0000000000002877 32649617 10.1097/TA.0000000000002877

[29] Pocock SJ, Vandenbroucke JP (2007) Strengthening the reporting of observational studies in epidemiology (StroBE) statement: guidelines for reporting observational studies. BMJ 223:806–808. 10.1136/bmj.39335.541782.ad 10.1136/bmj.39335.541782.AD PMC2034723 17947786

[30] Kokotovic D, Jensen TK (2023) Acute abdominal pain and emergency laparotomy: bundles of care to improve patient outcomes. Br J Surg 110:1594–1596. 10.1093/bjs/znad224 37449877 10.1093/bjs/znad224

[31] Tolstrup MB, Jensen TK, Gögenur I (2023) Intraoperative Surgical Strategy in Abdominal Emergency surgery. World J Surg 47:162–170. 10.1007/s00268-022-06782-9 36221004 10.1007/s00268-022-06782-9

[32] Kokotovic D, Burcharth J (2023) Enhanced recovery after emergency laparotomy. Br J Surg 110:538–540. 10.1093/bjs/znad056 36896630 10.1093/bjs/znad056

[33] Jensen TK, Burcharth J (2022) Strategies for open abdomen. Ugeskr Laeger 184(4):V08210640 35088692

[34] Gormsen J, Kokotovic D, Burcharth J, Jensen TK (2024) Standardization of the strategy for open abdomen in nontrauma emergency laparotomy: a prospective study of outcomes in primary versus temporary abdominal closure. Surgery 176(4):1289–1296. 10.1016/j.surg.2024.07.005 39122595 10.1016/j.surg.2024.07.005

[35] Harris PA, Taylor R, Minor BL et al (2019) The REDCap consortium: building an international community of software platform partners. J Biomed Inf 95:103208. 10.1016/j.jbi.2019.103208 10.1016/j.jbi.2019.103208 PMC7254481 31078660

[36] Todd B (2017) New CDC Guideline for the Prevention of Surgical Site infection. Am J Nurs 117:17. 10.1097/01.NAJ.0000521963.77728.c0 28749874 10.1097/01.NAJ.0000521963.77728.c0

[37] Dindo D, Demartines N, Clavien PA (2004) Classification of surgical complications: a new proposal with evaluation in a cohort of 6336 patients and results of a survey. Ann Surg 240:205–213. 10.1097/01.sla.0000133083.54934.ae 15273542 10.1097/01.sla.0000133083.54934.ae PMC1360123

[38] DeBord J, Novitsky Y, Fitzgibbons R et al (2018) SSI, SSO, SSE, SSOPI: the elusive language of complications in hernia surgery. Hernia 22:737–738. 10.1007/s10029-018-1813-1 30203373 10.1007/s10029-018-1813-1

[39] Center for Disease control and prevention (2024) Surgical Site Infection Event (SSI). https://www.cdc.gov/nhsn/pdfs/pscmanual/9pscssicurrent.pdf, accessed 16/09/2024

[40] Coccolini F, Roberts D, Ansaloni L et al (2018) The open abdomen in trauma and non-trauma patients: WSES guidelines. World J Emerg Surg 2:137. 10.1186/s13017-018-0167-4 10.1186/s13017-018-0167-4 PMC5797335 29434652

[41] Jensen TK, Nielsen YW, Gögenur I, Tolstrup M-B (2022) Sarcopenia is associated with increased risk of burst abdomen after emergency midline laparotomy: a matched case–control study. Eur J Trauma Emerg Surg 48(5):4189–4196. 10.1007/s00068-022-01958-3 35353215 10.1007/s00068-022-01958-3

[42] Kenig J, Richter P, Lasek A et al (2014) The efficacy of risk scores for predicting abdominal wound dehiscence: a case-controlled validation study. BMC Surg 14:1–6. 10.1186/1471-2482-14-65 25182865 10.1186/1471-2482-14-65 PMC4159378

[43] Khorgami Z, Shoar S, Laghaie B et al (2013) Prophylactic retention sutures in midline laparotomy in high-risk patients for wound dehiscence: a randomized controlled trial. J Surg Res 180:238–243. 10.1016/j.jss.2012.05.012 22677612 10.1016/j.jss.2012.05.012

[44] Mäkelä JT, Kiviniemi H, Juvonen T, Laitinen S (1995) Factors influencing wound dehiscence after midline laparotomy. Am J Surg 170:387–390. 10.1016/S0002-9610(99)80309-2 7573734 10.1016/s0002-9610(99)80309-2

[45] Webster C, Neumayer L, Smout R et al (2003) Prognostic models of abdominal wound dehiscence after laparotomy. J Surg Res 109:130–137. 10.1016/S0022-4804(02)00097-5 12643854 10.1016/s0022-4804(02)00097-5

[46] Söderbäck H, Gunnarsson U, Martling A et al (2019) Incidence of wound dehiscence after colorectal cancer surgery: results from a national population-based register for colorectal cancer. Int J Colorectal Dis 34:1757–1762. 10.1007/s00384-019-03390-3 31501927 10.1007/s00384-019-03390-3

[47] Jensen KK, Oma E, van Ramshorst GH et al (2022) Abdominal wound dehiscence is dangerous: a nationwide study of 14,169 patients undergoing elective open resection for colonic cancer. Hernia 26:75–86. 10.1007/s10029-020-02350-z 33394254 10.1007/s10029-020-02350-z

[48] Van Ramshorst GH, Nieuwenhuizen J, Hop WCJ et al (2010) Abdominal wound dehiscence in adults: development and validation of a risk model. World J Surg 34:20–27. 10.1007/s00268-009-0277-y 19898894 10.1007/s00268-009-0277-y PMC2795859

[49] Walming S, Angenete E, Block M et al (2017) Retrospective review of risk factors for surgical wound dehiscence and incisional hernia. BMC Surg 17:1–6. 10.1186/s12893-017-0207-0 28222776 10.1186/s12893-017-0207-0 PMC5320761

[50] Goodenough CJ, Ko TC, Kao LS et al (2015) Development and validation of a risk stratification score for ventral incisional hernia after abdominal surgery: Hernia expectation rates in intra-abdominal surgery (the HERNIA project). J Am Coll Surg 220:405–413. 10.1016/j.jamcollsurg.2014.12.027 25690673 10.1016/j.jamcollsurg.2014.12.027 PMC4372474

[51] Fischer JP, Basta MN, Mirzabeigi MN et al (2016) A risk model and cost analysis of incisional hernia after elective abdominal surgery based on 12,373 cases: the case for targeted prophylactic intervention. Ann Surg 263:1010–1017. 10.1097/SLA.0000000000001394 26465784 10.1097/SLA.0000000000001394 PMC4825112

[52] Coccolini F, Biffl W, Catena F et al (2015) The open abdomen, indications, management and definitive closure. World J Emerg Surg 10:1–10. 10.1186/s13017-015-0026-5 26213565 10.1186/s13017-015-0026-5 PMC4515003

[53] Bruns BR, Ahmad SA, O’Meara L et al (2016) Nontrauma open abdomens: a prospective observational study. J Trauma Acute Care Surg 80:631–636. 10.1097/TA.0000000000000958 26808023 10.1097/TA.0000000000000958

[54] Jairam AP, López-Cano M, Garcia‐Alamino JM et al (2020) Prevention of incisional hernia after midline laparotomy with prophylactic mesh reinforcement: a meta‐analysis and trial sequential analysis. BJS Open 4(3):357–368. 10.1002/bjs5.50261 32057193 10.1002/bjs5.50261 PMC7260413

[55] Tansawet A, Numthavaj P, Techapongsatorn S et al (2020) Mesh position for hernia prophylaxis after midline laparotomy: a systematic review and network meta-analysis of randomized clinical trials. Int J Surg 83:144–151. 10.1016/j.ijsu.2020.08.059 32927135 10.1016/j.ijsu.2020.08.059

[56] Indrakusuma R, Jalalzadeh H, van der Meij JE et al (2018) Prophylactic mesh reinforcement versus Sutured Closure to Prevent Incisional hernias after Open Abdominal aortic aneurysm repair via midline laparotomy: a systematic review and Meta-analysis. Eur J Vasc Endovasc Surg 56:120–128. 10.1016/j.ejvs.2018.03.021 29685678 10.1016/j.ejvs.2018.03.021

