# Supplementary material for: Short-term outcomes in mesh versus suture-only treatment of burst abdomen: a case-series from a university hospital
Source: Hernia. 2025 Feb 18;29(1):100. doi: 10.1007/s10029-025-03279-x (PMC11835968; doi:10.1007/s10029-025-03279-x)
Supplement: Supplementary file 1 — Supplementary Material 1 [file 10029_2025_3279_MOESM1_ESM.docx]

Supplemental table 1

| **Gender characteristics**  **N, total = 67(%)** | |  | | | |  |
| --- | --- | --- | --- | --- | --- | --- |
|  |  | **Male** | | **Female** | |  |
|  |  | **n = 47** | | **n = 20** | | ***P*** |
| Age (years) | <60 | 8 | (17) | 3 | (15) | .949^a^ |
|  | 60-80 | 31 | (66) | 14 | (70) |  |
|  | >80 | 8 | (17) | 3 | (15) |  |
| Body Mass Index (kg/m2) | ≥30 | 11 | (23) | 3 | (15) | .439^a^ |
| ASA-score | ≥ 3 | 20 | (43) | 8 | (40) | .846 |
| WHO performance status | ≥ 3 | 5 | (11) | 4 | (21) | .267^a^ |
| Index procedure | Emergency | 39 | (83) | 18 | (90) | .460 |
|  | Peritonitis^b^ | 20 | (43) | 8 | (40) | .846 |
| Type | Gastro-duodenal | 2 | (4) | 0 | (0) | .435^a^ |
|  | Adhesiolysis | 4 | (8.5) | 4 | (20) |  |
|  | Small bowel surgery^c^ | 15 | (32) | 7 | (35) |  |
|  | Large bowel surgery^c^ | 26 | (55) | 9 | (45) |  |
| Surgery for burst abdomen | Peritonitis^d^ | 10 | (21) | 7 | (35) | .237 |
|  | Course of open abdomen | 16 | (34) | 2 | (10) | .042^a^ |
|  | Conservative treatment | 4 | (9) | 3 | (15) | .418^a^ |

*ASA, American Association of Anesthesiologist; WHO, World Health Organization; CDC, Center for Disease Control and Prevention ^a^Fisher’s exact test
^b^ defined as* CDC wound classification grade III + IV *^c^ bowel resection, enterotomies and stoma creation*
